# Modern Corneal Eye-Banking Using a Software-Based IT Management Solution

**DOI:** 10.1155/2018/2645280

**Published:** 2018-05-20

**Authors:** C. Kern, K. Kortuem, C. Wertheimer, O. Nilmayer, M. Dirisamer, S. Priglinger, W. J. Mayer

**Affiliations:** ^1^Department of Ophthalmology, University Hospital LMU, Munich, Germany; ^2^Moorfields Eye Hospital, London, UK

## Abstract

**Background:**

Increasing government legislation and regulations in manufacturing have led to additional documentation regarding the pharmaceutical product requirements of corneal grafts in the European Union. The aim of this project was to develop a software within a hospital information system (HIS) to support the documentation process, to improve the management of the patient waiting list and to increase informational flow between the clinic and eye bank.

**Materials and Methods:**

After an analysis of the current documentation process, a new workflow and software were implemented in our electronic health record (EHR) system.

**Results:**

The software takes over most of the documentation and reduces the time required for record keeping. It guarantees real-time tracing of all steps during human corneal tissue processing from the start of production until allocation during surgery and includes follow-up within the HIS. Moreover, listing of the patient for surgery as well as waiting list management takes place in the same system.

**Conclusion:**

The new software for corneal eye banking supports the whole process chain by taking over both most of the required documentation and the management of the transplant waiting list. It may provide a standardized IT-based solution for German eye banks working within the same HIS.

## 1. Introduction

A global survey showed that keratoplasty is the most common tissue transplantation in the world, with 184.576 performed procedures involving 283.530 grafts in 2016 [1]. The regulations for the processing of human corneal grafts, which are regulated as pharmaceutical products, have increased in Germany within the last years because of new European and national regulations. In 2007, new legislation on the Quality and Safety of Human Tissues and Cells (Tissue law) incorporating amended European directives was passed by the German government. This resulted in major changes in medical law, transplant law and drug law. To define and control the quality requirements of donor tissue, corneal grafts were classified as a drug and not a human transplant. Therefore, this tissue is now regulated not only by tissue and transplant law, but also by drug law. The changes in the law generated increased documentation, processing costs and more thorough tests [2]. All regulations were reviewed and summarized by the “Deutsche Ärztekammer” (German medical council) and the “Paul-Ehrlich Institute” and led to new national guidelines [3, 4]. Because of the change in the laws, we adapted quality management and other regulations at our Cornea Bank accordingly to meet the new criteria [5]. As most of the documentation during processing at our tissue bank was paper-based or relied on the manual input of data into software such as Microsoft Excel, we observed an increase in the documentation time required during the processing of corneal grafts.

In 2013, to improve patient-related documentation at the university eye clinic, we started to develop a custom-made electronic health record (EHR) adjusted to the needs of ophthalmology based on the hospital information system (HIS) i.s.h.med (Cerner AG, Erlangen, Germany). This system is now used for the entire clinical documentation at our hospital [6, 7]. Using a Picture Archiving and Communication System (PACS), we linked diagnostic data from diagnostic devices to the clinical data of patients and built a data-warehouse including clinical and diagnostic data from more than 350,000 patients [8].

The processing of corneal grafts is documented on quality-audited paper forms. Manual repetitive documentation and manual data transfer between the eye hospital and the eye bank risk processing errors and do not guarantee a high safety level or auditability. Efficient, complete and comprehensible record-keeping is listed as one of the key features of eye banks in the literature [9].

Special requirements for electronic documentation were defined by the Bavarian Medical Council: safety of the network against access from outside, daily data and documentation back-up, long-term data storage, assignment of all entries to a responsible employee and, of course, proof of later additional changes in the patient's documentation [10]. These requirements can be achieved and guaranteed by using an established, professionally secured and backed-up HIS [11].

The aim of this project was to develop custom-made documentation algorithm based on our HIS, not only to improve documentation, but also to link waiting list management with transplant allocation. These data could enrich clinical information of importance concerning the number and quality of performed corneal transplantations at our clinic and should additionally simplify data analysis.

## 2. Material and Methods

### 2.1. Process Analysis

As a first step, the documentation process during corneal graft processing, together with waiting list management and allocation, was analysed and transferred into a process flow chart to guarantee the proper implementation and functionality of the new custom-made documentation software. Time measurements before and after introduction of the new system were recorded for three different steps: initial documentation on arrival of the donor tissue at the eye bank, documentation of nutritional liquid change of the graft and documentation for final clearance.

### 2.2. Software Requirements

Many requirements had to be considered before the development process was initiated. First, the software had to meet the criteria for documentation as defined in the “Good manufacturing practice” guideline provided by the Paul-Ehrlich Institute [3]. This involves the legibility and the auditability of the documentation. All changes in the documentation must be clearly visible and linked to the working user. Moreover, according to national guidelines, all documentation must be stored for at least 30 years after the expiry date of the product. These requirements are met by the well-established hospital IT platform run by the university hospital's IT department [12]. Access to the software must be limited for normal users as they should not be aware of links between donor and receiver identities.

Another requirement was the support of the employees of the eye banks during the documentation process, as most of their time was now used for documentation and not processing. In addition to the automatically performed timestamps, user logging, audit trails and lot numbers of the used material, the software must simplify the documentation process itself through pre-allocation.

### 2.3. Software Implementation

The development of the necessary user interface was performed within our HIS (i.s.h.med, Cerner AG, Erlangen, Germany), which is based on a SAP (SAP SE, Walldorf, Germany) platform by using Advanced Business Application Programming (ABAP) programming language [13]. The development process started in the late summer of 2016, with a first version being available in a HIS testing environment in spring 2017 during which functionality and stability were assessed. After several revisions to the software, the system was launched in December 2017.

## 3. Results

### 3.1. Process Analysis

Every single documentation step of the old process was identified and a data input structure was developed to define the requirements of the custom-made information input algorithm.

**Figure 1:**
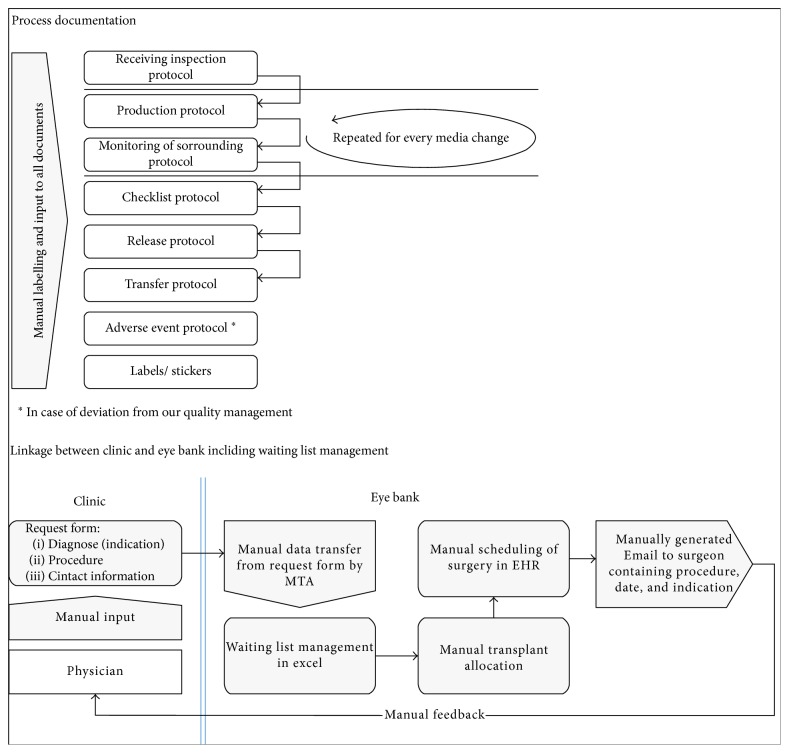
Process analysis before implementation of new software solution.

Several quality-managed Word files (Microsoft Corporation, Redmond, USA) were necessary for the monitoring and documenting of processing in the old workflow (Figure 1). After printing one set of documents per graft, a medical technical assistant (MTA) manually filled in the ongoing process documentation on paper. Once the processing was completed, the paper documents were archived in a folder and stored in an office.

The previous processing documentation and the listing and transplant allocation process were analysed (Figure 1). In the past, the waiting list was managed by using Microsoft Excel Spreadsheets (Microsoft Corporation, Redmond, USA) for which no audit trail was available. The whole process therefore needed to be optimized at every step and was subsequently defined in a detailed development plan of the new software.

### 3.2. Software Implementation

**Figure 2:**
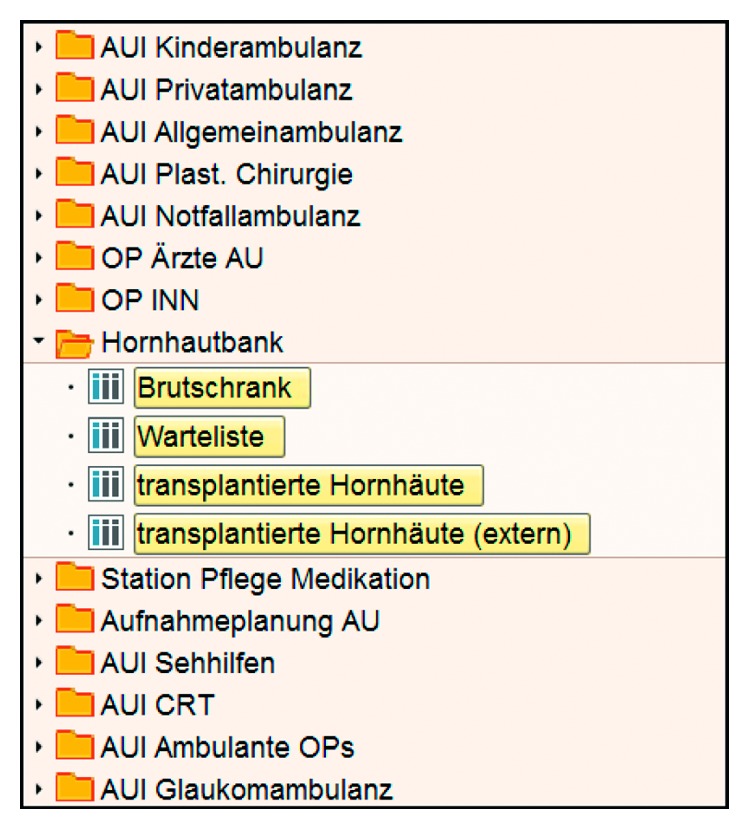
New eye bank working environment within the clinical HIS user interface. Translations related to corneal grafts only: Hornhautbank = Eyebank; Brutschrank = transplants in production; Warteliste = Waiting list; transplantierte Hornhäute = history of transplanted grafts; transplantiert Hornhäute (extern) = history of externally obtained transplanted grafts.

The software was implemented in the hospital's HIS by creating a new working environment for the eye bank and contained three different views: an overview of the processed transplants, the waiting list and a history of transplanted grafts for both our own and externally provided grafts (Figure 2). Based on assigned access rights, only eye bank employees can access the new working environment. All data are stored in the hospital's professionally managed redundant data centre, which guarantees data protection, high availability and regular data back-ups. Real-time data is stored on six different servers and weekly back-ups of the whole database are stored on servers using redundant array of independent disks technology (RAID-servers). Moreover, the data containing graft information are stored in the data-warehouse of the clinic to simplify data access for research and statistics.

### 3.3. Documentation of the Tissue-Processing Process

**Figure 3:**
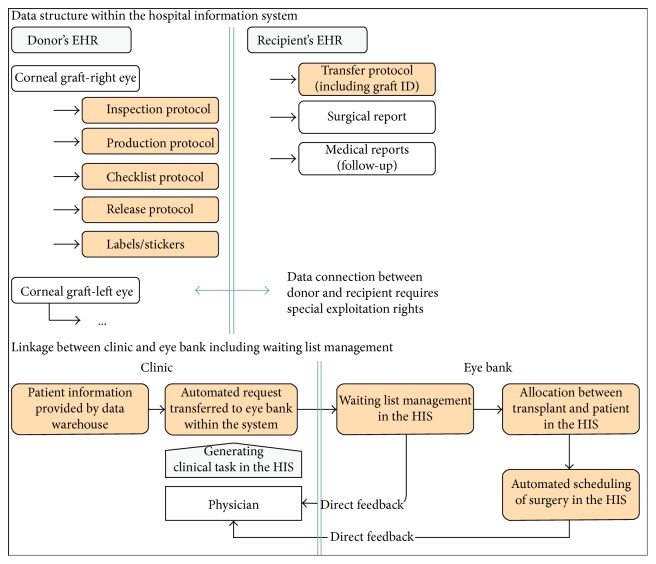
Process analysis after implementation of new software. All documents and steps shaded in peach are supported by the new software.

**Figure 4:**
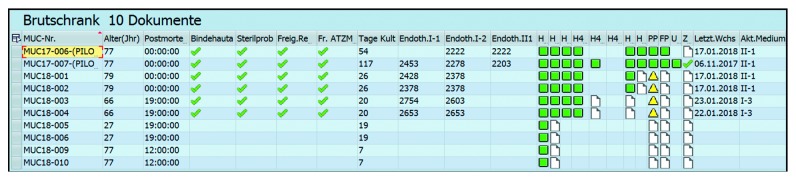
Working environment “Transplants in production.” Headings of columns starting on the left side: Graft ID, age donor, time between donor's death and explantation of the corneal graft, microbiological testing of conjunctiva, microbiological testing of first medium, clearance by forensic medicine, clearance by serological testing, number of days cultivated, endothelial count I-1, I-2 and II-1, status of documents (blank sheets means the document can be set up, a yellow triangle symbolizes a document without final clearance (changes still possible) and the green square indicates a finished document), last medium change, actual medium.

Five new user interfaces (HIS-term: parametric medical documents (PMD)) have been developed to allow process documentation. All PMDs are linked to a patient within the HIS patient master index. Therefore, all donors are registered, if they have not been a patient during their lifetime, in the category “cornea donor.” This categorization allows data access to be limited to those employees with special access rights. The processing protocol including the graft ID is linked to the recipient's EHR. This allows the linking of graft ID and the donor's identity for eye bank staff (special access rights) in cases of any adverse events. Graft and surgery complications are recorded in the recipient's EHR and can be queried by the eye bank staff at any time from the data warehouse.  Figure 3 shows the data structure of the new processing documentation process. In a university setting, final clearance for all grafts is provided by a responsible consultant with the necessary access rights. Follow-up documentation within the recipient's EHR is guaranteed by this data structure, even though, due to information governance issues, backtracking of the graft's origin is not possible for the treating physician. To provide a better overview of multiple processed transplants, all data appear in a view in the working environment (Figure 4). This contains the graft's ID, the donor's information and clearance following various microbiological tests.

### 3.4. Linkage between Clinic and Eye Bank

**Figure 5:**
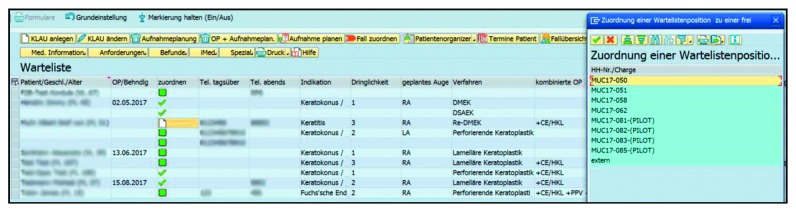
Working environment “waiting list.” The process of linking a patient from the waiting list to a graft is shown. Headings of the columns starting on the left: patient/sex/age if available, scheduled surgery, connection, daytime phone number, evening phone number, indication, side, procedure, combined procedure. The column headed connection shows a green tick if the surgery has been performed, a green square if it is scheduled and a blank sheet of paper if no connection between this patient and a graft exists. Clicking on the blank paper, you can see a selection of the available grafts from the working environment of “transplants in production.”

Improvements in the listing of patients for corneal transplantation and in the feedback loop were further objectives of this software solution. A new item in the entry management part of the HIS was developed to enable the safe and rapid listing of patients for the physician. The clinical and contact information of the patient is automatically added to data fields before the inclusion of the patient to the waiting list. This step replaces the manually filled-in request form (Figure 3). After the allocation of a transplant to a recipient, a new surgical procedure in the theatre diary will be generated providing the surgeon with direct feedback about the date, time and relevant procedure. Relevant parameters about the graft are provided to the surgeon in the operation theatre on an automatically filled in checklist, which is printed and attached to the graft package. The whole waiting list management takes now place within the user interface of the HIS (Figure 5). To provide a transparent system of allocating tissues in the waiting list, we defined three different urgency groups based on clinical criteria: “elective,” “urgent,” “emergency.” For the first group, grafts are provided depending on the patients' overall waiting time, for the second group within 4 weeks and an emergency listed patient will receive the next available transplant. After a processed transplant has been matched to a patient on the waiting list, all stickers and forms are generated automatically by using previously entered data. After the removal of the patient from a waiting list position, the transplanted cornea is moved to a third subfolder of the eye bank environment, where information about the donor, the processing process and the recipient is visualized. This enables employees with the necessary rights to obtain a rapid overview of donors, corneal graft IDs, recipients, indications and the date and type of surgery. Data can be also filtered by date to simplify annual statistics for internal or official use.

### 3.5. Time Measurements

Before the introduction of the new system, the time consumed during initial documentation was 30 minutes per graft. Changing the nutritional liquid took 20 minutes and final clearance 60 minutes of documentation time. With the new system, initial documentation time decreased to 10 minutes per graft. Changing the graft liquid only took 5 minutes and the final clearance 20 minutes of documentation per graft. This equals a reduction of 66% for initial documentation, 75% for liquid change and 66% for final clearance.

## 4. Discussion

After the analysis of the existing process and the definition of certain documentation requirements as legally specified, new software has been implemented to streamline our corneal graft processing. The new software features a high degree of automation and supports the linkage of clinics and our eye bank. Compared with former paper-based and hand-written documentation, many tasks have been automated (e.g., time, date and lot numbers). The implementation within the leading HIS has made a transparent and clear tissue allocation process with the highest amount of information being available to all involved staff to guarantee patient safety. All processing and transplant allocation data are now safely stored on hospital servers according to German/European data safety and storage guidelines [14].

The generation of a waiting list order position via a patient EHR is one of the key features of the new software. It guarantees easy access from every clinic desk and immediate listing during clinical examination. The automatic addition of each patient's personal and clinical data into the list should eliminate errors during the listing of patients. Moreover, this system simplifies the workflow as the generation of a list position within the EHR replaces the previously used paper-based versions. Subjectively, the data quality within EHR-based listing form has increased through the new system by providing all the necessary clinical information and the correct contact information. After a request for a graft by a physician, the eye bank employee receives immediate feedback through the sending of the clinical task to the worklist as explained by Kortuem et al. [7]. Within the HIS, comments can be added and waiting list positions can be edited and, thus, all information is clearly visible to managing employees.

In 2016, a survey was performed by the “Deutsche Ophthalmologische Gesellschaft” (German ophthalmologic society). It covered questions related to IT infrastructure at eye hospitals. The results have shown that the most commonly used system is i.s.h. med in 13 German eye hospitals [11]. Worldwide, more than 500 hospitals participate in Cerners' HIS solution [15]. Developed as a module of i.s.h. med, our eye bank software can easily be transferred to other clinics by using the same HIS. This will expand the usability spectrum of the systems at other clinics.

Recently, a corneal transplant registry was set up at India's National Eye Bank including the follow-up data of graft recipients. According to the authors, the database simplified data collection for follow-up compared with former paper-based outcome analysis [16]. The connection of the corneal graft documentation process to the EHR simplifies access to data regarding the processing of the tissue and the graft itself [8, 11]. This provides further helpful information about the graft to the surgeon prior surgery. Within the existing data-warehouse, graft-specific data can be easily matched to the clinical data of the follow-up examinations of the recipients. This provides easy access to data for further statistical analysis, quality control, research, recipient follow-up and the correlation of graft data to clinical outcome and possible complications.

Many legal and professional regulations exist concerning the safety assessment of donor and recipient electronic records [10]. In addition to tracking changes in the records made by users and the identification of changes in the existing documentation, network safety must be guaranteed [14]. The used HIS should however provide regular back-up and long-term data storage and, moreover, the servers running the system should receive clearance from a data protection officer [11]. By implementing the software within an established HIS, all mandatory points concerning data protection and auditability have been met. Furthermore, in cases of any required inspections by the responsible authorities, access to the whole documentation can be granted through a digital system.

By the development of a custom-made documentation software into out HIS, we improved the dataflow between the clinic and eye bank, with a saving of 66% of documentation time. Electronic documentation systems can thus reduce the workload for employees and even increase patient safety because of the automatic, less error-prone transfer of data between the clinic and tissue bank.

## Data Availability

The data used to support the findings of this study are available from the corresponding author upon request.
